# Ginsenoside Rb1 Mitigates Myocardial Fibrosis Through Inhibiting Exosomal‐Derived miRNA‐21–Associated Inflammation and Cardiac Signaling

**DOI:** 10.1155/cdr/8873721

**Published:** 2026-03-26

**Authors:** Shihua Wang, Weifeng Chi, Yinghong Lu, Guangyu Yu, Jie Li, Yingying Nie, Jingxiang Zhou, Yanjie Song, Wenyan Ji

**Affiliations:** ^1^ Department of Cardiology, Qingdao Hiser Hospital Affiliated of Qingdao University (Qingdao Traditional Chinese Medicine Hospital), Qingdao, China

**Keywords:** angiotensin II, exosome, ginsenoside Rb1, miRNA-21, myocardial fibrosis

## Abstract

Myocardial fibrosis exemplifies a crucial pathological event in the escalation of heart failure. Natural bioactive agents that can modulate and mitigate myocardial fibrosis hold promising potential as key therapeutic and preventive strategies for managing heart failure. In this study, Ginsenoside Rb1 was found to attenuate myocardial fibrosis and suppress inflammatory responses by downregulating exosomal miRNA‐24 expression in the experimental mice models. Primary mouse cardiac fibroblasts (pMCFs) were isolated from neonatal C57BL/6J mice for in vitro assays. For the in vivo investigations, adult C57BL/6J mice were utilized to assess the effects of Rb1 and Ang II on exosome‐derived inflammatory pathways. Multiple analytical techniques, including qPCR, Western blotting, nanoparticle tracking analysis (NTA), BrdU staining, and ELISA, were employed to evaluate gene expression, protein levels, exosome characterization, cell proliferation, and cytokine secretion. The obtained results showed that Rb1 prevents Ang II–induced cell death and BrdU‐positive cells in the pMCFs. Moreover, we have confirmed that the exosomes derived from pMCF cells have a size range of 80–250 nm, with a peak at 149 nm. RBb‐1 prevented the Ang II–exposure‐stimulated exosomal mRNA and protein expression of cardiac fibrosis markers (COL1a1, COL3a1, ACTA2, and TGF*β*1) in both pMCFs and mice models. Moreover, Rb1 inhibited the inflammatory expression which was confirmed by ELISA. In addition to that, Rb1 impedes the exosomal mRNA expression of miRNA‐21 in the mouse model. This study will open up a new insight into the relationship between Rb1 and miRNA‐21, which mitigates myocardial fibrosis in the Ang II–induced mouse model.

## 1. Introduction

Myocardial fibrosis (MF) is crucial for pathological remodeling and malfunction of the heart. Fibrosis plays a significant role in the underlying cause of the disease and is linked to almost all types of heart failure. Generally, cardiomyocytes have a limited ability to regenerate the damage to the heart causes cardiomyocyte death [1]. Heart failure and mortality are typically the outcome of excessive deposition of extracellular matrix (ECM) components in the heart, which can lead to various cardiovascular disorders such as cardiac fibrosis, hypertension, myocardial infarction, and cardiomyopathy [2]. Cardiovascular disease (CVD) is considered the main cause of death and has a significant negative impact on both health and the economy. CVD accounts for one‐third of all deaths globally, with an estimated 18 million deaths annually [3]. However, statistics indicate that by 2030, the rate of death is anticipated to increase to 24 million deaths annually, which means 42% of total global deaths [4]. Besides different mechanisms for fibrotic response, cardiomyocyte death and myocardial inflammation due to injury are the main causes of cardiac fibrosis. The relative contribution to fibrotic response was the excess secretion of ECM proteins by various cell types, such as macrophages, mast cells, lymphocytes, and vascular cells, which are also said to be fibrogenic mediators [5].

There are currently no viable treatments for cardiac fibrosis, and the only therapeutic option for individuals in late stages is heart transplantation, even this extreme procedure is made difficult by the scarcity of donors [6]. To develop novel antifibrotic medicines and reduce the significant morbidity and mortality associated with CVDs, numerous studies conducted in recent years have concentrated on improving our understanding of the cellular and molecular pathways participating in the CF activation process [2]. Numerous therapeutic applications, such as epigenetic therapies, miRNA inhibitors, and antifibrotic drugs, have been used in preclinical research [7]. miRNAs have been demonstrated to be crucial factors in regulating embryonic development in the cardiac tissues, and they might induce the pathogenesis of cardiac fibrosis–mediated myocardial infarction [8]. Subsequent investigation supports the theory that miR‐29 may target fibrosis‐related mRNAs that encode collagens, for example, Col1A1, MMPs, and integrins. Moreover, TGF*β* is a regulator of miR‐29 expression, resulting in cardiac fibrosis [9].

MicroRNA 21 (miR‐21) is a small regulatory RNA that is highly expressed and encoded by the locus 17q23.2 of the human genome. miR‐21 is expressed both intracellularly and extracellularly, with notable presence in numerous body fluids that contribute to MF [10]. miR‐21 plays a crucial role in protecting cardiomyocytes from cardiac stress in the initial stage. Later, it can stimulate pathological consequences of cardiac fibrosis by activating fibroblasts [11]. This behavior of miR‐21 has resulted in several diseases such as coronary artery disease, heart failure, cardiomyopathy, and myocardial infarction [12]. miR‐21 promoted cardiac fibrosis and inflammation through Smad7‐associated signaling in the experimental models [13]. Hence, inhibition of miR‐21–associated signaling is considered a novel approach to prevent MFs.

Ginsenosides are a class of triterpenoids that make up the majority of the fundamental chemical and pharmacological properties of Panax plants, including *Panax ginseng, Panax notoginseng, and Panax quinquefolius*. Generally, ginsenosides are categorized according to their natural abundance into primary saponins (ginsenoside Rb1, Rg1, Re, Rd, etc.) and secondary ginsenosides (Rg5, Rk1, Rg3, etc.) [14]. Ginsenoside, the main active ingredient in ginseng, has over 110 different chemical structural classifications and is known to have anti‐inflammatory, antioxidant, antiapoptotic, and antiautophagy effects on the neurological system [15]. The results of recent research suggested possible cardiovascular protective effects of RH4 by inhibiting Ang II–induced myocardial hypertrophy, inflammatory fibrosis, and oxidative stress which was also demonstrated by in vitro models [15]. Similarly, another study with Rb1 showed that pathological damage caused by aconitine can be prevented by Rb1 intervention, which suppresses the myocardial enzyme levels [16].

Exosomes, also known as extacellular vesicles (ECVs), play a role in various pathological processes. Exosomes directly communicate with cellular organelles and macromolecules, thereby regulating cellular signaling events in the cells [17]. Exosomes lie in their role as key mediators of intercellular communication that transmit profibrotic and pro‐inflammatory signals in cardiocytes [18]. Specifically, exosomes carry microRNAs, especially miR‐21, which are known to promote cardiac fibroblast activation, collagen synthesis, and TGF‐*β*–driven fibrosis [18]. Rb1 treatment–mediated prevention of cardiac fibrosis and inflammatory responses in the exosomes of cardiac tissues and cardiac fibroblast cells has not yet been elucidated. Therefore, in this study, we have investigated whether Rb1 could prevent angiotensin II–induced MF by targeting exosomal miRNA‐21–associated inflammation and cardiac biomarkers. Based on this, it is evident that exosomes can serve as potential diagnostic tools for CVD, as they carry several biomolecules that reflect myocardial pathological changes. Furthermore, the study demonstrates that the impact of Rb1 on MF can be effectively evaluated at the exosomal level, highlighting exosomal biomarkers as a valuable platform for assessing therapeutic responses.

## 2. Materials and Methods

### 2.1. Chemicals and Reagents

Angiotensin II and ginsenoside Rb, fetal bovine serum (FBS), collagenase, tetrazolium bromide, DAPI, and phosphate‐buffered saline (PBS) were purchased from Sigma‐Merck, United States. DMEM medium, Trypsin‐EDTA (0.05%), and penicillin–streptomycin, BrdU from ThermoFisher Scientific, United States. Primary monoclonal antibodies such as COL1A1 (#72026), COL3A1 (#66887), TGF‐*β*I (#49728), ACTA2 (#19245), and *β*‐actin (#4967) were purchased from cell signaling technology, Shanghai, China. All the primers were designed by prime blast, NCBI database. Primers were purchased from Eurofins, China. Other chemicals and reagents used in this study were molecular grade.

### 2.2. Experimental Model for In Vitro Studies

For isolating primary cardiac fibroblast cells, 1–3‐day‐old male C57BL/6J neonatal mice were purchased from Slack Animal Co., Ltd. The experiments were performed following international ethical guidelines and the National Institutes of Health “Guide for the Care and Use of Laboratory Animals”.

### 2.3. Isolation and Culture of Primary Cardiac Fibroblasts

The mice were rinsed with distilled water, followed by disinfection with 75% medical alcohol. The mice were subjected to anesthesia (ketamine injection) and were operated on surgically to harvest the heart from the chest cavity. The excised heart was immediately transferred into ice‐cold sterile HBSS buffer (without Ca^2+^ and Mg2+) and then cut into small pieces using a sterile blade. The tissue was then transferred into the Eppendorf tube, containing a digestion solution (0.08% Trypsin + 0.06% Type II collagenase). Digestion was performed in a water bath at 37°C for 10 min, and the same procedure was repeated five times until a clear upper suspension layer was discarded [19]. The obtained cell pellet was resuspended in 1 mL of HBSS buffer to terminate digestion. The cell mixture was centrifuged, and the resultant cell pellet was resuspended in DMEM medium (containing 10% FBS and 100 U/mL of penicillin/streptomycin) and incubated at 37°C.

### 2.4. MTT Assay

The 96‐well plate was inoculated with an equal number of Ang II (1 *μ*M) and Rb1 (3.125–100 *μ*M) treated cells and incubated for 48 h. Cells were washed with PBS after the incubation, and 50 *μ*L MTT reagent of concentration 0.5/mL was added. After 4 h of incubation, 100‐*μ*l DMSO was added to dissolve the formazan crystals. The purple color generated by the dissolution of formazan crystal is assessed calorimetrically using a microplate reader at 570 nm to determine the viability of the cells [20].

### 2.5. BrdU Staining Assay

The treated cells were labeled with BrdU, as mentioned in the manufacturer′s manual. BrdU incorporation was visualized by indirect immunofluorescence staining using anti‐BrdU antibodies and rhodamine‐labeled goat antimouse antibodies. DAPI (4 ^′^,6‐diamidino‐2‐phenylinodole) is used as a costain to visualize the nuclei of the cells [21].

### 2.6. In Vivo Experimental Models and Treatments

Adult male C57BL/6J mice were provided from Slack Animal Co., Ltd. The experimental environment was set up as mentioned in the following: The mice had access to adequate water and food. Also, the temperature and relative humidity were maintained at 23°C ± 2°C and 50%–60%, respectively. Light/dark cycles of 12 h were implemented. Adult male C57BL/6J mice were divided into five experimental groups, with each group consisting of six animals: (i) Control mice receiving 0.5% DMSO as vehicle control, (ii) myocardia fibrosis model mice prompted by Ang II group (1000 ng/kg/day), (iii) mice were treated with the low‐dose of Rb1 (6.25 mg/kg/b.wt) with Ang II, (iv) mice were treated with the medium‐dose of Rb1 (25 mg/kg/b.wt) with Ang II, and (v) mice were treated with the high‐dose of Rb1 (100 mg/kg/b.wt) with Ang II. Ang II was given by using a microosmotic pump (1002, Alzet, United States) [22]. The pump was entrenched subcutaneously in the back of mice and delivered 100 ng/kg/b.wt. Ang II was administered for 4 weeks. Rb1 treatments began 2 weeks after Ang II infusion and continued thereafter to the end. Rb1 was given by oral gavage administration. At the end of the experiments, mice were sacrificed, hearts were carefully harvested for several biochemical experiments.

### 2.7. Isolation of Exosomes From Cells and Cardiac Tissue

Exosomes were isolated from the conditioned medium of primary cardiac fibroblasts cultured in DMEM medium by using the total Exosome Isolation Kit (Umibio Biotechnology, China). The brief protocol was followed by Yuan et al. [23]. Exosome vesicles (EV) were isolated from the mouse cardiac tissues following the detailed method by Liang et al. [24]. After completion of treatment, the mice were euthanized using Fatal‐Plus (Pentobarbital Sodium, 100 mg/kg). The thoracic cavity was opened surgically, the right atrium was nicked to drain blood, the heart was excised, and it was immediately transferred into ice‐cold HBSS. Then, 2 mL of digestion buffer (HBSS supplemented with Liberase DH 200 *μ*g/mL and DNase I 20 U/mL) was added to a 35‐mm plate on ice, and 100–120 mg of fresh cardiac tissue was placed into the buffer. The tissues were minced into ~2 mm^3^ fragments and incubated at 37°C on an orbital shaker (50 rpm) for 45 min. After digestion, the tissue was gently pipetted up and down ~20 times using a P1000 pipette with the tip trimmed (∼2 mm) to prevent clogging. The suspension was filtered through a 70‐*μ*m cell strainer into a 50‐mL centrifuge tube, and the plate was rinsed with 5 mL of HBSS to collect any residual material. The filtrate was centrifuged at 300 × g for 10 min at 4°C to remove large debris, and the supernatant was transferred to a new tube without disturbing the pellet. The supernatant was centrifuged again at 2000 × g for 20 min at 4°C to eliminate smaller cells and apoptotic bodies. The resulting supernatant was filtered through a 0.8‐*μ*m syringe filter into an ultracentrifuge tube. Three milliliters of HBSS was added to prevent clogging, and the tubes were balanced. After the rotor was precooled for 12–18 h at 4°C, the supernatant was centrifuged at 16,500 × g for 25 min at 4°C to collect the large EV fraction (~200–800 nm). The supernatant was carefully decanted and subsequently centrifuged at 118,000 × g for 150 min at 4°C to pellet the small ECVs (~50–200 nm). The supernatant was discarded, the tubes were inverted briefly to drain residual liquid, excess moisture was blotted with a KimWipe, and the ECV pellet was resuspended in 500 *μ*L of ice‐cold PBS. Both ECV fractions were transferred to fresh microtubes and kept on ice until further use.

### 2.8. Nanoparticle Tracking Analysis (NTA)

The isolated exosomes were placed on the copper grid, and after the liquid was absorbed, the phosphotungstic acid solution was added dropwise on the copper grid along with PKH67 counter stain to observe the morphology of exosomes. To further understand the morphological properties of exosomes, such as particle size and concentration, NTA technology was used. The particle size and concentration of exosomes were measured by the Nanosight NS300, and NTA 3.0 software was used [25]. A group without exosomes was used as a blank control.

### 2.9. ELISA

The level of inflammatory markers like IL‐1*β*, IL‐6, and TNF‐*α* was analyzed by ELISA techniques. The 96‐well microtiter plate was precoated with antibodies specific to the inflammatory markers, and the sample was diluted and loaded. The plate was then washed at least two times after 4 h of incubation. Furthermore, the horseradish peroxidase (HRP)–conjugated antiantibodies specific to inflammatory markers (anti–IL‐1*β*, anti–IL‐6, and anti–TNF‐*α*) were added and incubated for an additional 2 h. After incubation, the plates were again washed thoroughly, and the substrate solution for HRP was added and incubated for 15 min. To terminate the reaction, the stop solution was added, and after the color changed to yellow, the colorimetric analysis was done by measuring the optical density at 450 nm using a microplate reader [26]. The result was finally analyzed by using the standard curve, and the dilution factor was also accounted.

### 2.10. qPCR

The exosomal mRNA from the exosomes was extracted by using a commercial kit (Invitrogen, United States). The isolated RNA was reverse transcribed into cDNA using the Prime Script RT Reagent Kit from ThermoFisher Scientific. Further, this cDNA was amplified with gene‐specific primers using the PowerUP SYBR Green Master Mix kit (ThermoFisher Scientific) to analyze the gene expression of cardiac fibrosis markers, namely, COL1A1, COL3A1, TGF*β*1, and ACTA2 [27]. The expression of miRNA‐21 is also analyzed by a similar method. The list of primers used in this study is shown in Table 1.

**Table 1:** List of primers.

S. No	Gene	FP	RP
1	COL1A1	ATGTGCCACTCTGACTGGAA	TCCATCGGTCATGCTCTCTC
2	COL3A1	CCCCTGGTTCTTCTGGACAT	TGGGCCTTTGATACCTGGAG
3	TGF‐*β*1	TCGCTTTGTACAACAGCACC	ACTGCTTCCCGAATGTCTGA
4	ACTA2	GCTATTCAGGCTGTGCTGTC	GGTAGTCGGTGAGATCTCGG
5	GAPDH	AGGTCGGAGTCAACGGATTT	TGACGGTGCCATGGAATTTG
6	MiRNA‐21	TAGCTTATCAGACTGATGTTGA	TGTCAGACAGCCCATCGACTGGTGTTGCCATGAGATTCAACAGTCAAC

### 2.11. Western Blot

The total exosomal protein from the exosomes was extracted by using a commercial kit (Invitrogen, United States). Protein concentration was measured using the Bradford technique. Proteins were separated by electrophoresis using 10% SDS‐PAGE and then placed onto nitrocellulose membranes with a thickness of 0.45 *μ*m. For 1 h, the membrane was blocked with 5% bovine serum albumin, and then it was incubated overnight at 4°C with the corresponding monoclonal antibodies specific to the protein of interest. Further, the membrane was washed three times, incubated for an hour at room temperature with secondary antibodies, and detected using a chemiluminescent detection device [27].

### 2.12. Statistical Analysis

Data are represented as mean ± standard deviation (SD) from three replicates. Analysis of variance (ANOVA) was used to differentiate among the multiple experimental groups. A statistical variance of *p* < 0.05 was measured as significant.

## 3. Results

### 3.1. Rb1 Prevents Ang II–Induced Cell Death in Primary Cardiac Fibroblast Cells

To investigate the Rb1 intervention in the Ang II–induced primary cardiac fibroblast cell viability, the cells were treated with Ang II (1 *μ*M) following that different doses of Rb1 (3.125, 12.5, 25, 50, and 100 *μ*M) for 48 h and analyzed for cell viability using the MTT assay. The percentage of cell viability in each group was calculated and plotted against the concentration of Rb1. The result depicted that Ang II stimulation increased cell death in primary cardiac fibroblasts. However, Rb1 increasing treatment started with 3.125–100‐*μ*M concentration following Ang II (1 *μ*M) intervention significantly reduced the cell death in a dose‐dependent manner. The high‐dose Rb1 group showed a maximum of 90% cell viability (Figure 1a). Hence, we found that Rb1 prevents Ang II–stimulated cell death in primary cardiac fibroblast cells. We also imaged (Figure 1b) the morphological changes caused by Rb1 intervention at low (50 *μ*M) and high (100 *μ*M) Rb1 dosages.

Figure 1Rb1 prevents Ang II–induced cytotoxicity in primary cardiac fibroblast cells. (a) Rb1 and Ang II–mediated cytotoxicity was evaluated by MTT‐based cytotoxicity assay. Data are represented as mean ± standard deviation (SD) from three replicates. Analysis of variance (ANOVA) was used to differentiate among the multiple experimental groups. A statistical variance of *p* < 0.05 was measured as significant. (b) Microscopic images of primary cardiac fibroblasts treated with Ang II and Rb1.

**Figure:**
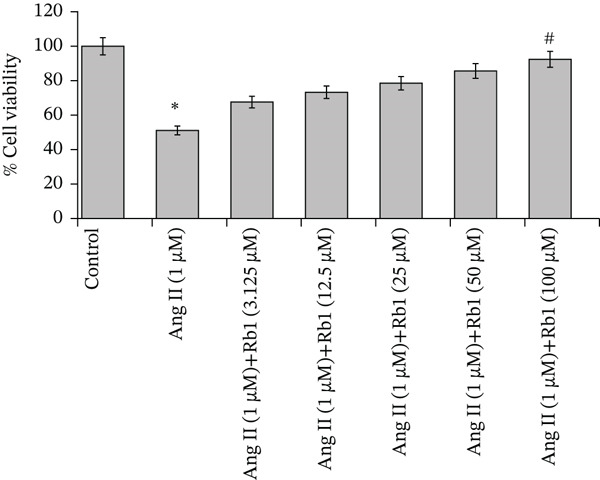
(a)

**Figure:**
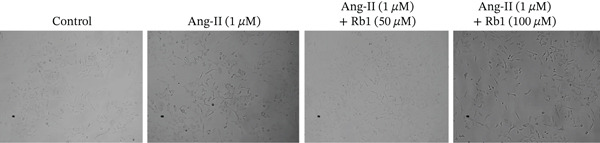
(b)

### 3.2. Rb1 Reduced Ang II–Induced BrdU Positive Cells in Cardiac Fibroblast Cells

Figure 2 represents the results of Rb1 treatment on Ang II–induced BrdU‐positive nuclei proliferation in primary cardiac fibroblasts. The results obtained showed that exposure to Ang II alone enhanced the number of BrdU‐positive nuclei, causing MF. However, Rb1 treatment prevented the Ang II–induced BrdU‐positive nuclei proliferation in primary cardiac fibroblasts. Hence, Rb1 has the ability to reduce cardiac fibrosis induced by Ang II.

Figure 2Rb1 reduced Ang II–induced BrdU‐positive cells in cardiac fibroblast cells. (a) Representative images show BrdU (red) staining, DAPI (blue) staining merged with both red and blue. (b) Quantification of BrdU‐positive cells in cardiac fibroblast cells. Data are represented as mean ± standard deviation (SD) from three replicates. Analysis of variance (ANOVA) was used to differentiate among the multiple experimental groups. A statistical variance of *p* < 0.05 was measured as significant.

**Figure:**
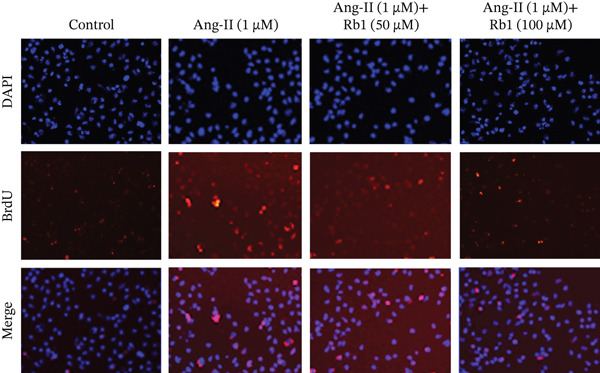
(a)

**Figure:**
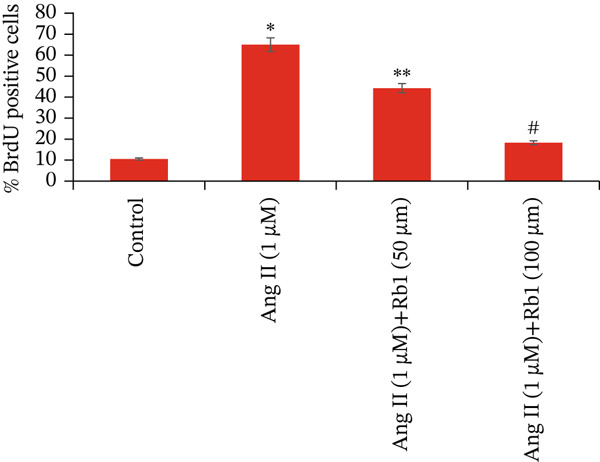
(b)

### 3.3. Exosome Particle Size and Concentration Were Identified by Using Nanoparticle Tracking Analyzer

The particle size of the isolated exosome ranged from 80–250 nm with the peak at 149 nm. Using NTA, the particle size distribution of the isolated exosome was also determined to be 0.944. This study confirmed that the exosome isolated displayed good morphological characteristics. (Figure 3a,b).

Figure 3Characterization of exosomes. (a) Exosome particle size analyzed DLS and the particle size was ranged from 80–250 nm with the peak at 149 nm. (b) Concentration was identified by using nanoparticle tracking analyzer.

**Figure:**
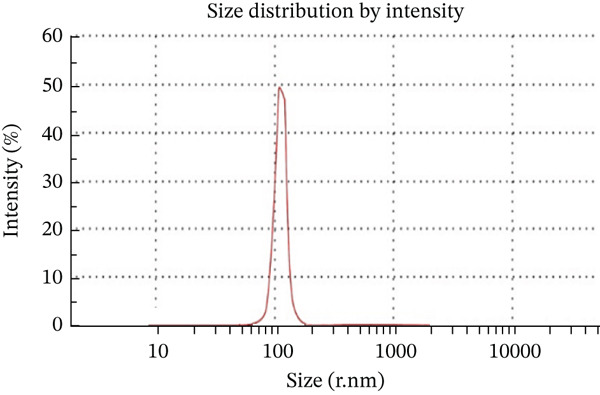
(a)

**Figure:**
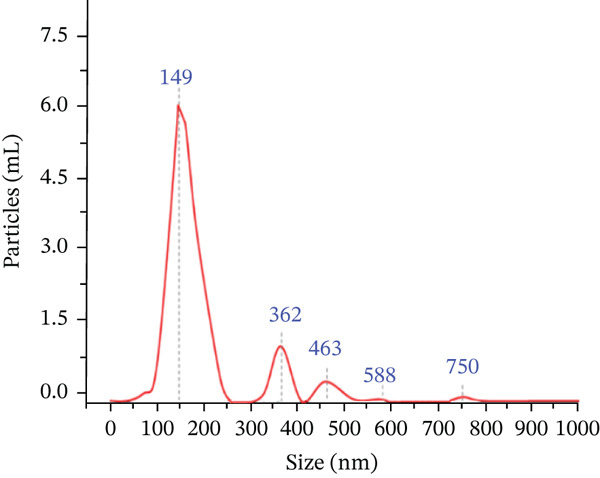
(b)

### 3.4. Rb1 Inhibits the Expression of Cardiac Fibrosis Markers (COL1a1, COL3a1, ACTA2, and TGF*β*1) in Primary Cardiac Fibroblast Cell–Derived Exosomes

The effect of Rb1 in Ang II stimulated cardiac fibrosis was analyzed by checking the expression of cardiac fibrosis markers such as COL1a1, COL3a1, ACTA2, and TGF*β*1 in primary cardiac fibroblast cell–derived exosomes. Figure 4a,b shows the RT‐PCR analysis stated that Ang II stimulation caused significant upregulation of COL1a1, COL3a1, ACTA2, and TGF*β*1 mRNA expressions; whereas subsequent intervention of Rb1 downregulated the mRNA expression of COL1a1, COL3a1, ACTA2, and TGF*β*1 in the fibroblast exosomal sample. Moreover, Rb1 in Ang II stimulated cardiac fibrosis marker protein expression assessed by western blotting (Figure 4c,d). Rb1 treatment prevented Ang II–induced the overexpression of cardiac fibrosis markers COL1a1, COL3a1, ACTA2, and TGF*β*1 in primary cardiac fibroblast cell–derived exosomes.

Figure 4Rb1 inhibits the expression of cardiac fibrosis markers in primary cardiac fibroblast cells derived exosomes. (a) RT‐PCR studies for Rb1 on Ang II–stimulated cardiac fibrosis mRNA genes such as COL1a1, COL3a1, ACTA2, and TGF*β*1 in primary cardiac fibroblast cells derived exosomes. (b) Densitometric analysis for COL1a1, COL3a1, ACTA2, TGF*β*1, and GAPDH was used to normalized equal loading RNA samples. (c) Western blot analysis for Rb1 on Ang II–stimulated cardiac fibrosis protein expressions of COL1a1, COL3a1, ACTA2, and TGF*β*1 in primary cardiac fibroblast cells derived exosomes. (d) Densitometric analysis for COL1a1, COL3a1, ACTA2, TGF*β*1 and *β*‐actin was used to normalize equal loading protein samples. Data are represented as mean ± standard deviation (SD) from three replicates. Analysis of variance (ANOVA) was used to differentiate among the multiple experimental groups. A statistical variance of *p* < 0.05 was measured as significant.

**Figure:**
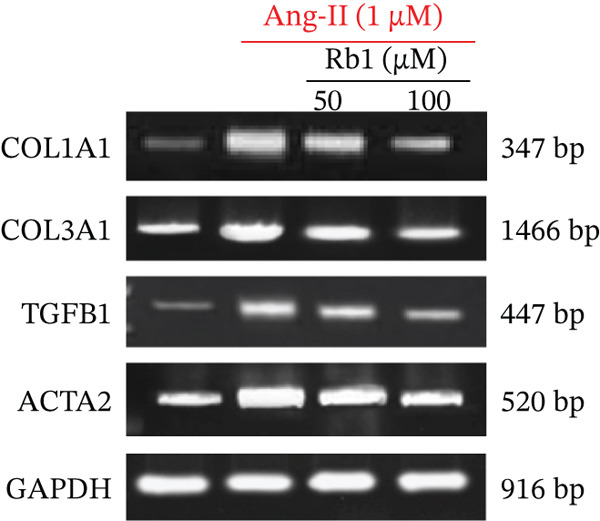
(a)

**Figure:**
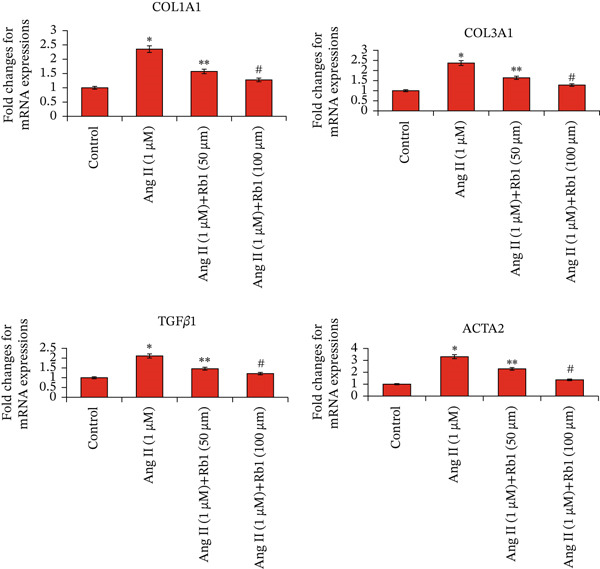
(b)

**Figure:**
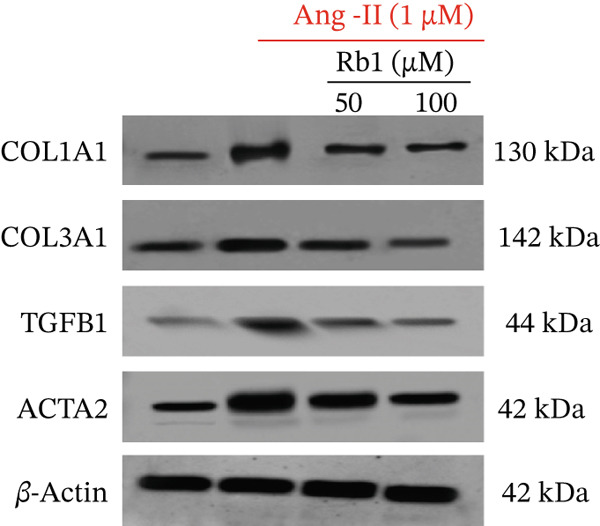
(c)

**Figure:**
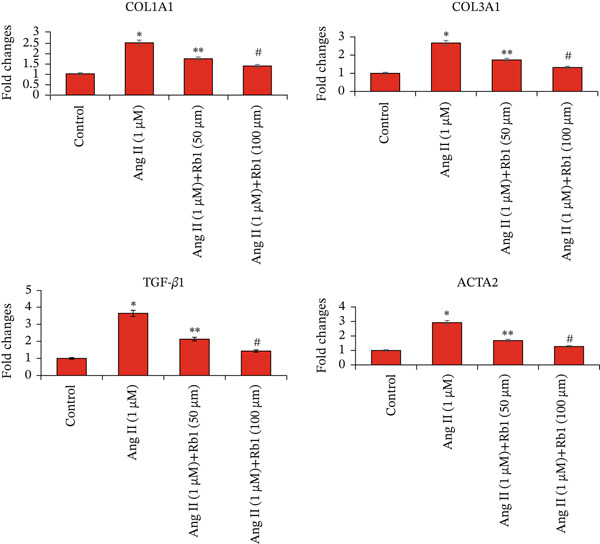
(d)

### 3.5. Rb1 Inhibits the Expression of Inflammatory Factors Such as IL‐1*β*, IL‐6, and TNF‐*α*

Overexpression of inflammatory genes involved enhances the pathogenesis of MF. The effect of Rb1 on Ang II stimulated inflammatory genes in the primary cardiac fibroblast cells derived from exosomes was evaluated by ELISA (Figure 5). Exosomes from Ang II treated primary cardiac fibroblast cells show significant enhancement of inflammatory genes such as IL‐1*β*, IL‐6, and TNF‐*α*. However, Rb1 low doses and high doses with Ang II treated cells significantly reduced the expression of IL‐1*β*, IL‐6, and TNF‐*α* in the exosomal samples.

Figure 5Rb1 inhibits the expression of inflammatory genes in primary cardiac fibroblast cells derived exosomes. The expression of IL‐*β*1 (a), IL‐6 (b) and TNF‐*α* (c) in primary cardiac fibroblast cells derived exosomes were evaluated by ELISA. Data represented as mean ± Standard deviation (SD) from three replicates. Analysis of variance (ANOVA) was used to differentiate among the multiple experimental groups. A statistical variance of *P* < 0.05 was measured as significant.

**Figure:**
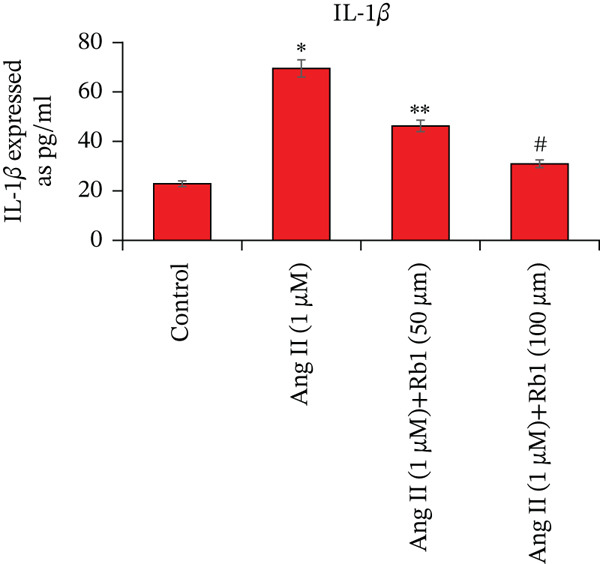
(a)

**Figure:**
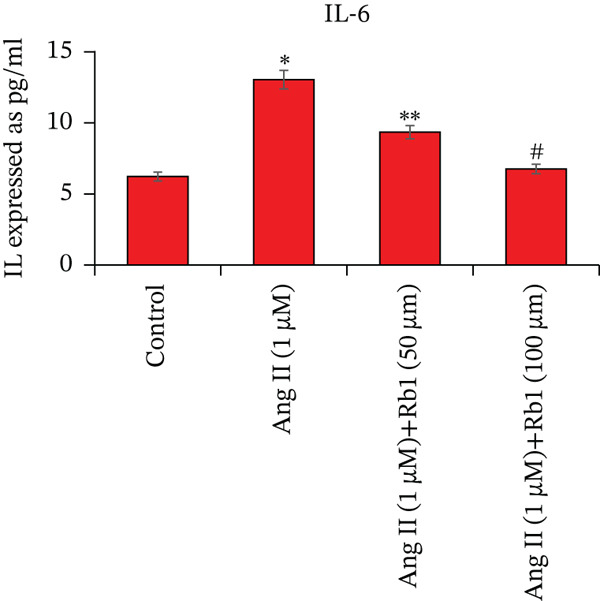
(b)

**Figure:**
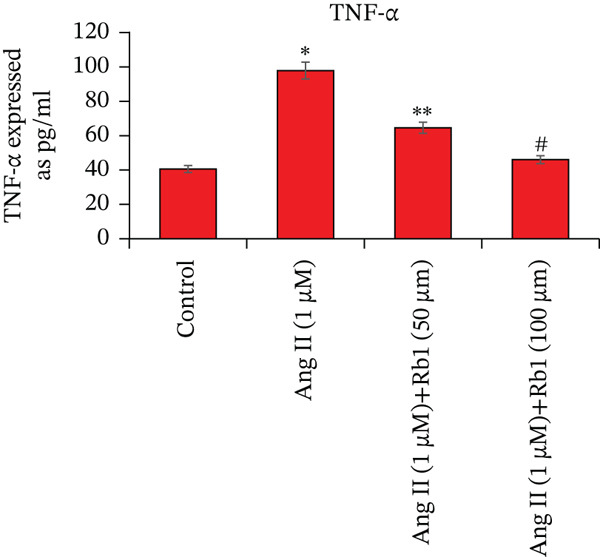
(c)

### 3.6. Rb1 Suppress Ang II–Induced Cardiac Fibrosis Marker Expressions in the Mouse Cardiac Tissue–Derived Exosomes

First, we have investigated the effect of Rb1 on Ang II–stimulated cardiac fibrosis in primary cardiac fibroblast cell–derived exosomes. To confirm whether the same effects occur in in vivo experiments, the expression study was done with the exosomal fraction of protein as well as mRNA to examine whether the Rb1 intervention inhibits the expression of cardiac fibrosis marker on the mice cardiac tissue–derived exosome. The RT‐PCR results showed that when Ang II stimulation was applied, it significantly increased the mRNA expression of cardiac fibrosis indicators on the surface of the exosome, such as COL1A1, COL3A1, TGF*β*1, and ACTA2 (Figure 6a,b). Rb1 significantly downregulated Ang II–induced mRNA expression of cardiac fibrosis markers COL1A1, COL3A1, TGF*β*1, and ACTA2 in the mice cardiac tissue–derived exosomes.

Figure 6Rb1 inhibits the expression of cardiac fibrosis markers in mice tissue–derived exosomes. (a) RT‐PCR studies for Rb1 on Ang II–stimulated cardiac fibrosis mRNA genes such as COL1a1, COL3a1, ACTA2, and TGF*β*1 in mice tissue derived exosomes. (b) Densitometric analysis for COL1a1, COL3a1, ACTA2, TGF*β*1, and GAPDH was used to normalized equal loading RNA samples. Data represented as mean ± standard deviation (SD) from three replicates. Analysis of variance (ANOVA) was used to differentiate among the multiple experimental groups. A statistical variance of *p* < 0.05 was measured as significant.

**Figure:**
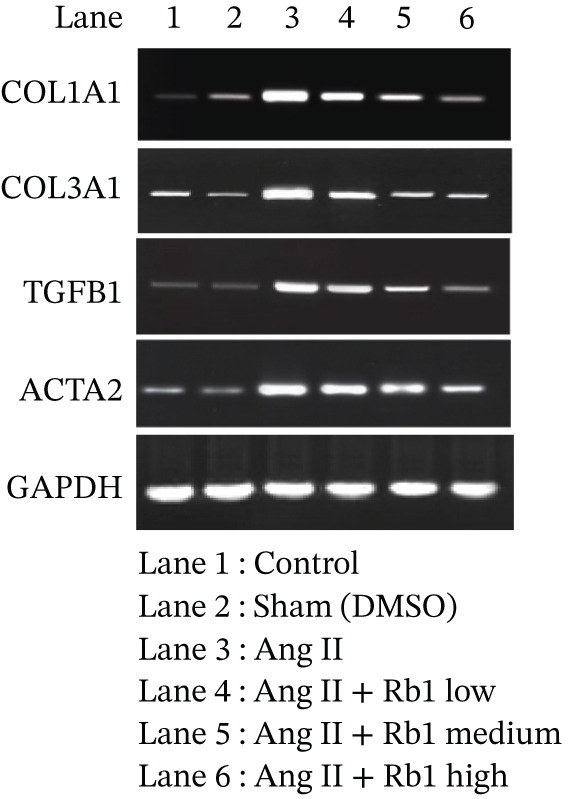
(a)

**Figure:**
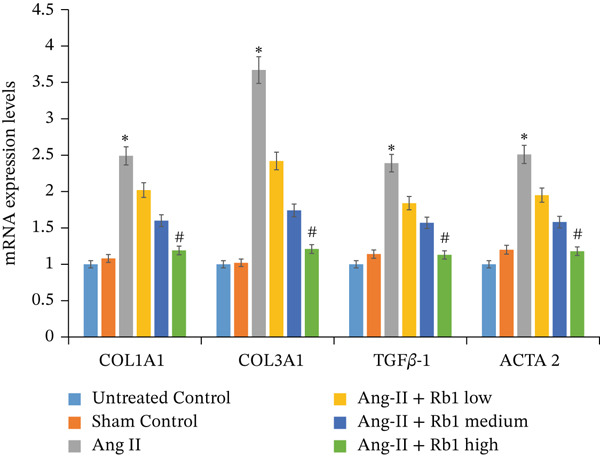
(b)

In addition, Rb1 on Ang II–induced protein expression of cardiac markers COL1A1, COL3A1, TGF*β*1, and ACTA2 in the mice cardiac tissue–derived exosomes was studied by Western blotting (Figure 7a,b). Rb1 treatment also suppressed Ang II–induced protein expression of cardiac fibrosis markers COL1A1, COL3A1, TGF*β*1, and ACTA2 in the mice cardiac tissue–derived exosomes. The Rb1‐mediated inhibition of MF was dose‐dependent manner.

Figure 7Rb1 inhibits the expression of cardiac fibrosis markers in mice tissue–derived exosomes. (a) Western blot analysis for Rb1 on Ang II–stimulated cardiac fibrosis protein expressions of COL1a1, COL3a1, ACTA2, and TGF*β*1 in mice tissue–derived exosomes. (b) Densitometric analysis for COL1a1, COL3a1, ACTA2, TGF*β*1, and *β*‐actin was used to normalize equal loading protein samples. Data are represented as mean ± standard deviation (SD) from three replicates. Analysis of variance (ANOVA) was used to differentiate among the multiple experimental groups. A statistical variance of *p* < 0.05 was measured as significant.

**Figure:**
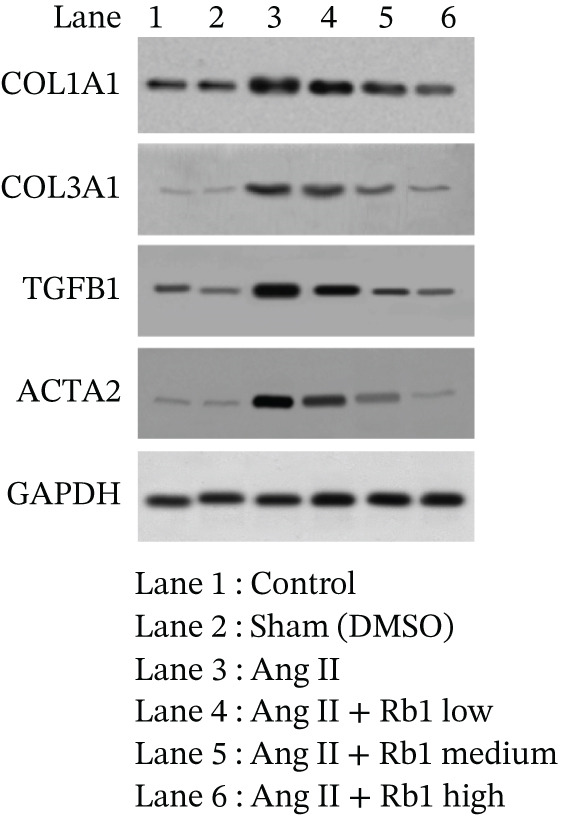
(a)

**Figure:**
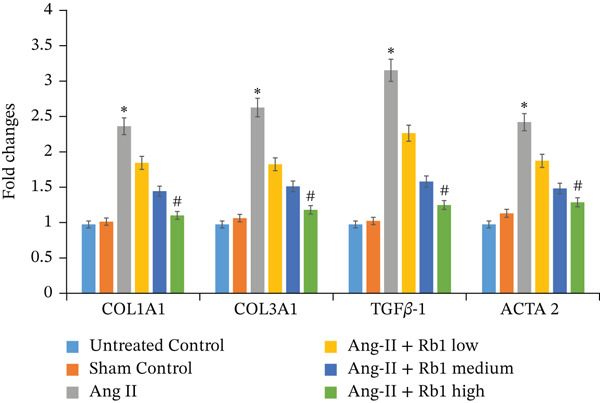
(b)

### 3.7. Rb1 Impedes miRNA‐21 Expression in Ang II–Induced Cardiac Fibroblast Cells

**Figure 8:**
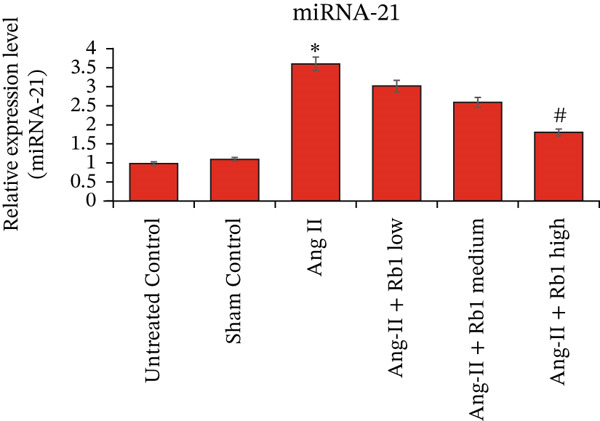
Rb1 inhibits the expression of miRNA‐21 in mice tissue–derived exosomes. RT‐PCR studies for Rb1 on Ang II–stimulated miRNA‐21 in mice tissue derived exosomes. GAPDH was used normalized equal loading RNA samples. Data are represented as mean ± standard deviation (SD) from three replicates. Analysis of variance (ANOVA) was used to differentiate among the multiple experimental groups. A statistical variance of *p* < 0.05 was measured as significant.

The combined results of all the experiments performed suggested that Rb1‐mediated cardiac fibrosis suppression is regulated by miRNA‐21. To investigate this concept, the expression of miRNA‐21 in the mice cardiac tissue–derived exosomes is done by RT‐PCR. The results supported the concept, where higher expression of miRNA‐21 was observed in the Ang II group when compared with all other groups (Figure 8). The Rb1 intervention with three different dosages showed a gradual reduction in the expression of miRNA‐21, which interprets that Rb1 suppresses cardiac fibrosis by downregulating the expression of miRNA‐21.

## 4. Discussion

MF is one of the main reasons for the development of numerous CVDs, including myocardial infarction. Even though the novel technologies help to understand the pathophysiology of cardiac fibrosis, there are no proven therapeutic drugs to cure or prevent cardiac fibrosis [27]. The current methods for diagnosing cardiac fibrosis are either invasive or need specialized imaging. Generating and identification of a circulating biomarker would eliminate many of these challenges. Recent investigations have highlighted the significance of exosomal contents, particularly miRNAs, and their usage as a possible biomarker [27]. Hence, this study investigated ginsenoside Rb1 to prevent angiotensin II–induced MF by targeting exosomal miRNA‐21–associated inflammation and cardiac biomarkers. Further, the phytochemical intervention in the cardiac fibrosis treatment field is blooming with novel or well‐established phytochemical agents. Ginsenosides are potent phytochemical agents that are well known for anticancer and antineuro disorders activity. Curcumin and ginsenosides have been reported to have anti‐inflammatory properties against intestinal cell types and proved that it is a useful therapeutic agent to lessen radiation‐induced damage [28]. One of ginseng′s most significant active ingredients is ginsenoside Rb1, which has numerous studies of research supporting its cardioprotective potential [29].

Cardiac fibrosis has a significant role in the chemical, mechanical, and electrical signaling of the heart, and disruption of these signaling pathways can lead to cardiac dysfunction. Numerous kinds of conditions, such as myocardial stiffness, atrial fibrillation (AF), and insufficient oxygen and nutritional distribution, can cause fibrosis that negatively impacts heart function [30]. The result of the MTT assay depicted that Ang II stimulation increased cell death of cardiac fibroblasts by 50%, but the Rb1 intervention significantly reduced the cell death in a dose‐dependent manner. The high‐dose Rb1 group showed a maximum of 90% cell viability. Previously, Rb1 significantly decreased the cell death of cardiac fibroblasts due to Ang II toxicity [29].

The major manifestation of cardiac fibrosis is said to be the increased extracellular deposition of collagen due to the upregulating certain genes like COL1a1 and COL3a1 [31]. TGF*β*1 is also an important profibrotic cytokine which activates fibrosis via the Smad3, PI3K/Akt, and MAPK signaling pathways [31]. The gene expression studies proved that Rb1 intervention significantly reduced the expression of cardiac fibrosis exosomal markers such as COL1A1, COL3A1, TGF*β*1, and ACTA2. Many strong research evidences also suggested the involvement of miRNA‐21 in cardiac fibrosis particularly in the stimulation of collagen synthesis, fibroblast survival, and growth factor release [32]. As recent studies were highly focused on exosome‐mediated therapeutics for cardiac fibrosis, we also investigated the expression of cardiac fibrosis marker on the exosome which showed similar results. A study outcome proved that in a rat model of MI, injection of human peripheral blood‐derived exosomes loaded with a miRNA‐21 inhibitor, which is rich in exosomes derived from cardiac fibroblasts decreased cardiac fibrosis [33].

miRNA‐21 promotes the development of fibrosis by altering the dynamics of the ECM, controlling fibroblast alterations, and modulating signaling pathways [34]. This has been proved by BrdU staining which showed that Rb1 treated cells inhibit the proliferation when compared with Ang II group cells. This result also suggested that Rb1 intervention reduces the cardiac fibroblast proliferation. Since the inflammatory response is a major determinant in cardiac fibrosis, we focused on the expression of inflammatory factors due to Rb1 intervention, which in turn controls the inflammatory response. According to preclinical evidence, exosomes can downregulate the circulating inflammatory markers and macrophage mobilization [35]. The same has been observed in ELISA, which showed that the level of inflammatory markers like IL‐1*β*, IL‐6, and TNF‐*α* downregulated by the intervention of Rb1.

We investigated the miRNA target for Rb1 that can be known for its cardioprotective potential. The result that the expression of miRNA‐1 and miRNA‐2280 was increased due to the effect of Rb1 and in contrast, the expression of miRNA‐21 and miRNA‐320 was downregulated by Rb1 [29]. Our study also confirmed that higher expression of miRNA‐21 was observed in the Ang II group when compared with all other experimental groups. The Rb1 intervention with three different dosages showed a gradual reduction in the expression of exosomal miRNA‐21, which interprets that Rb1 suppresses cardiac fibrosis by downregulating the expression of miRNA‐21. Previously, hyperoside, a natural drug, prevents sepsis‐associated cardiac dysfunction by inhibiting the growth of cardiomyocytes and inflammatory responses via inhibiting miR‐21 [36].

## 5. Conclusion

This study exemplifies that Ginsenoside Rb1 efficiently mitigates Ang II–stimulated MF and inflammation by modulating exosome‐mediated molecular pathways. Rb1 not only protected cell viability and reduced fibrotic marker expression in primary cardiac fibroblasts but also attenuated fibrosis markers and inflammatory cytokine secretion in vivo models. The inhibitory effect of Rb1 on exosomal miRNA‐21 expression further highlights a novel regulatory mechanism underlying its cardioprotective action. Collectively, these findings provide new insights into the therapeutic potential of Rb1 as a natural bioactive compound capable of targeting exosome‐associated profibrotic signaling, thereby offering a promising strategy for preventing or treating myocardial fibrosis in heart failure.

## Author Contributions

**S.W.** and **W.C.**: conceptualization and original draft preparation; **Y.L.** and **G.Y.**: methodology; **J.L., Y.N.,** and **J.Z.**: data curation, **Y.S.** and **W.J.**: supervision and original draft preparation. **Y.S.** and **W.J.** contributed equally to this work.

## Funding

This study was supported by the Shandong Provincial Natural Science Foundation (ZR2023QH160) and Qingdao Natural Science Foundation (23‐2‐1‐146‐zyyd‐jch).

## Disclosure

All authors agreed to publish this paper in this journal. All authors read and approved the final version of the manuscript.

## Ethics Statement

The animal research procedures were approved by the Medical College of Qingdao University (Ethics Approval Number QDU‐AEC‐2022441) and carried out in accordance with the Guide for the Care and Use of Laboratory Animals (Ministry of Science and Technology of China, 2006).

## Consent

The authors have nothing to report.

## Conflicts of Interest

The authors declare no conflicts of interest.

## Data Availability

The data that support the findings of this study are available from the corresponding authors upon reasonable request.
